# A Frequency-Selective Reconfigurable Antenna for Wireless Applications in the S and C Bands

**DOI:** 10.3390/s23218912

**Published:** 2023-11-02

**Authors:** Alexandros Sakkas, Vasilis Oikonomou, Giorgos Mystridis, Vasilis Christofilakis, Giorgos Tatsis, Giorgos Baldoumas, Vasilis Tritiakis, Spyridon K. Chronopoulos

**Affiliations:** 1 Electronics-Telecommunications and Applications Laboratory, Physics Department, University of Ioannina, 451 10 Ioannina, Greece; a.sakkas@uoi.gr (A.S.); pph100776@uoi.gr (V.O.); pph100803@uoi.gr (G.M.); gtatsis@uoi.gr (G.T.); gbaldoumas@uoi.gr (G.B.); 2 Mariolopoulos-Kanaginis Foundation for the Environmental Sciences, 106 75 Athens, Greece; vas@mariolopoulosfoundation.gr

**Keywords:** reconfigurable antenna, PIN diode, patch antenna, measurements, multifrequency

## Abstract

This paper presents a compact multifrequency reconfigurable patch antenna in terms of design and fabrication for operating in the S and C bands of the RF spectrum, which are overwhelmed by wireless applications. Reconfiguration is achieved by using a single PIN diode on the ground plane. By varying the voltage applied to the diode, three modes can emerge, exhibiting main resonant frequencies at 2.07, 4.63, and 6.22 GHz. Resonance switching requires a voltage of less than 0.9 V. The antenna fabricated on an FR-4 substrate, with a volume of 70 × 60 × 1.5 mm^3^, has a radiating patch element of a rectangular ring shape. The proposed low-cost antenna is easily implemented in a typical university lab-based environment. The total bandwidth for the three modes is close to 1 GHz, while the voltage standing wave ratio (VSWR) of the fabricated version of the antenna does not exceed 1.02, and the return loss is well below −40 dB for the three primary resonant frequencies.

## 1. Introduction

The rapid growth of wireless communications has recently led to the demand for platforms supporting several communication standards. Such platforms can benefit highly from reconfigurable antennas, which can function in multiple bands, radiation patterns, or polarizations. Reconfigurable antennas are currently used in cognitive radio systems, satellite communications, MIMO systems, biomedical applications, as well as military and industrial applications [1,2]. The process of designing them can be tedious because of their complexity. Achieving optimal results is challenging for different antenna characteristics (return loss, gain, impedance matching, etc.) while ensuring that these results remain satisfying for all modes of operation. Also, depending on the reconfiguration mechanism used, several factors need to be considered, such as the systems used to provide the required biasing of electrical elements, the way they affect the antenna’s performance, the power consumption, the switching times, etc. [3,4]. Despite the design difficulties, there is a strong interest in reconfigurable antennas due to their attractive benefits. Their multifunction capabilities, easily adjustable via dedicated software, coupled with their small size, lack of filtering element requirements, high isolation, etc., make them excellent candidates for future applications [5].

Reconfigurable antennas can change their resonant frequencies, radiation patterns, polarization, or any combination thereof. Reconfiguration is achieved usually by one of the following methods: electrical switching, optical switching, mechanical switching, and smart materials.

Reconfiguration via electrical switching is the most common method used. It involves PIN (positive–intrinsic–negative) diodes, varactor diodes, or RF MEMS (radiofrequency micro-electromechanical system). Optical switching concerns using photoconductive switches, while mechanical switching involves the physical movement of radiating parts [3,6]. Finally, “smart materials” that can alter their characteristics, like graphene, liquid crystals, liquid metals, etc., can also be used for reconfigurable antennas [7].

Many researchers have presented simulation results of reconfigurable antennas of various geometries [8]. In some cases, only the proposed antenna is also implemented. The authors of [9] simulated and implemented a reconfigurable antenna with a flower-shaped patch, operating in the WLAN and WiMAX bands. Their frequency switching relied on a single varactor diode, and the effect of altering the diode’s positioning was tested. An antenna using two PIN diodes is presented in [10], covering several frequencies from 3.1 to 9.5 GHz, while changing the radiation pattern for different operation modes. A multiband frequency reconfigurable antenna was designed and implemented in [11]. Three PIN diodes were placed on the ground plane providing the switching mechanism for frequencies between 1.36 and 8.6 GHz. In [12], the authors used six PIN diodes on a pixel antenna, achieving three configuration modes with resonant frequencies at 2.6, 3.9, and 10 GHz. A reconfigurable MIMO antenna using RF MEMS switches is introduced in [13] with resonances from 800 MHz to 5.5 GHz. In [14], a frequency and pattern reconfigurable antenna is presented. A single PIN diode provided two modes, resonating at (1) 2.47 and 5.36 GHz, or (2) 3.8 GHz. Another frequency and pattern reconfigurable antenna proposed in [15] is based on three PIN diodes. The radiating element is a double-open-ring patch, and the resonant frequencies lie between 1.9 and 5.6 GHz. A reconfigurable antenna that can rotate its radiation pattern between −36° and +36° is presented in [16]. The antenna operates in the 5 GHz band, and the pattern reconfiguration is achieved by a feeding network using sixteen PIN diodes. The authors of [17] introduce a polarization reconfigurable antenna operating in the 2–3 GHz region. By varying the capacitance of four varactor diodes using a voltage up to 7.5 V, the polarization changes between right- or left-handed circular, horizontal, and vertical. Another polarization reconfigurable antenna switching between right-hand and left-hand circular polarization based on two PIN diodes is presented in [18], being appropriate for 5 G applications in the 3.5 GHz band. A phase change material, Germanium Telluride (GeTe), is used for a polarization reconfigurable antenna in [19] for applications in the 30 GHz band. By irradiating ultraviolet (UV) short laser pulses, the material switches between an insulating (OFF) and a metallic (ON) state. As a result, the antenna’s polarization transitions between linear and circular, right or left-handed. An intriguing device, for absorption and conversion of electromagnetic energy, is presented in [20]. It is based on a four-ring multi-resistance unit, and it can be used as an energy harvester, converting microwave energy into thermal energy and eventually electrical energy. Its efficiency at 5.8 GHz is 99.5%. Finally, a frequency reconfigurable antenna, intended for cognitive radio applications, is shown in [21]. It operates on a wide band between 2.63 and 3.7 GHz, and it utilizes 12 PIN diodes on the ground plane.

Most of the available literature concerning antenna reconfiguration characteristics via PIN diodes has so far concentrated on two or more PIN diodes [10,11,12,15,18,21,22,23,24,25,26]. On the other hand, fewer studies have been published with only one PIN diode [14,27,28]. In the present work, a single PIN diode greatly simplifies the design, the implementation, and the cost of the antenna. At the same time, the experimental results show a compact reconfigurable antenna with three dominant resonance frequencies at 2.07, 4.63, and 6.22 GHz and a wide bandwidth range where several wireless protocols, including IoT, are crowded into these frequency bands. Furthermore, such antenna technology could have a huge effect on military applications. Imagine a UAV/USV (unmanned aerial/surface vehicle) that incorporates a large number of small printed reconfigurable antennas on its structure that could act as an array of antennas, without or even with symmetry, exhibiting profound effects such as the ability to scan a beam with low alteration in either the beam width or the side lobe level. Additionally, a phased array could contain a considerable number of reconfigurable antennas. So, there is a boosted ability of an electronically controlled scheme, while creating a smart-radio beam that could be steered toward different directions and with different frequencies without physical movement. Considering the aforementioned in relation to the simplicity of implementing the suggested antenna, we can understand the impact on long and short communications (LoRa and SRC).

The presented antenna was designed for integration into a high-precision signal power measurement setup, akin to the one detailed in [29]. This setup serves the purpose of gathering data about rain-induced attenuation. The investigation of rain rate through signal attenuation measurements has garnered significant interest in recent years [30]. To facilitate portability and effortless placement within the experimental framework, a compact antenna was imperative. Moreover, its multifrequency attributes are essential for comprehensively studying attenuation concerning rain rate across multiple frequencies. The switching mechanism of the antenna which can be easily controlled by a microcontroller is also valuable for this application. 

This paper is organized as follows. Section 2 describes the antenna geometry. Simulation results are presented in Section 3. Experimental results and discussion are presented in Section 4, followed by the conclusions in Section 5.

## 2. Antenna Geometry

**Figure 1:**
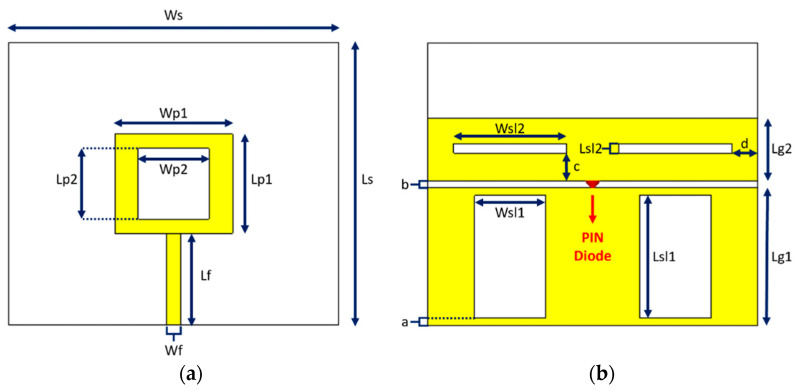
Structural geometry of the antenna (**a**) Front view, (**b**) Back view.

The structural geometry of the antenna is illustrated in Figure 1. The antenna printed on an FR-4 material exhibits a dielectric constant of 4.3 and a height of 1.5 mm. The copper’s height is 35 μm. The radiating element is a rectangular ring-shaped patch. The antenna is excited via a microstrip feed line. The ground plane, on the bottom side of the antenna, consists of two rectangular parts, and the PIN diode is placed in the middle between them, with its anode being on the top. Four rectangular slots were also inserted on the ground plane in places where secondary undesired resonances showed maximum surface currents, reducing or canceling their effect. The dimensions of the slots, as well as those of the patch, substrate, and ground plane, were adjusted to achieve optimal results. The final value for each design parameter appears in Table 1.

## 3. Simulation Results

**Figure 2:**
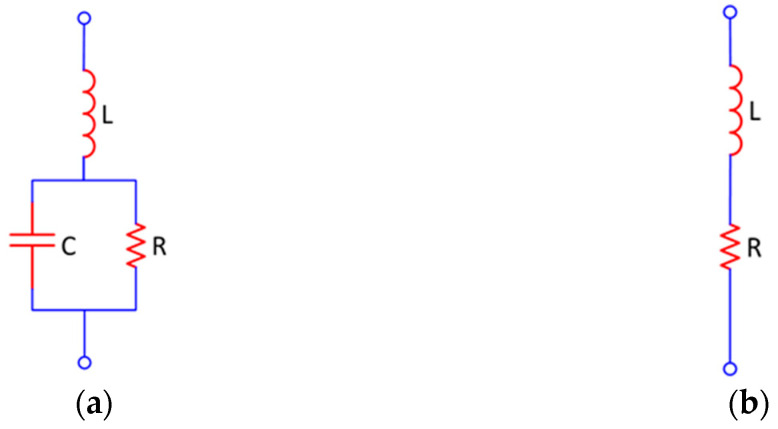
The equivalent circuit of PIN diode when (**a**) reverse-biased, and (**b**) forward-biased.

The antenna simulation was conducted using CST Studio Suite 2019 with open-space boundary conditions. To provide excitation, a waveguide port was positioned at the edge of the microstrip feed line, with a port extension coefficient of 7.2. For the substrate material, lossy FR-4 was used, complemented by parts of annealed copper. The model of the PIN diode used is Skyworks SMP1302 in an SC-79 package. According to its datasheet, the diode can be used in applications from 10 MHz to beyond 10 GHz. In the simulation, the diode had to be represented by its equivalent circuit, because the simulation tool does not support nonlinear elements. The diode can be represented by an RL circuit when it is forward-biased (ON state) or by an RLC circuit when reverse-biased (OFF State), as shown in Figure 2. In the case of forward biasing, it acts as a current-controlled resistor, while in the case of reverse biasing, it acts as a capacitor in parallel with a high-value resistor. Inductance depends on the package used.

**Figure 3:**
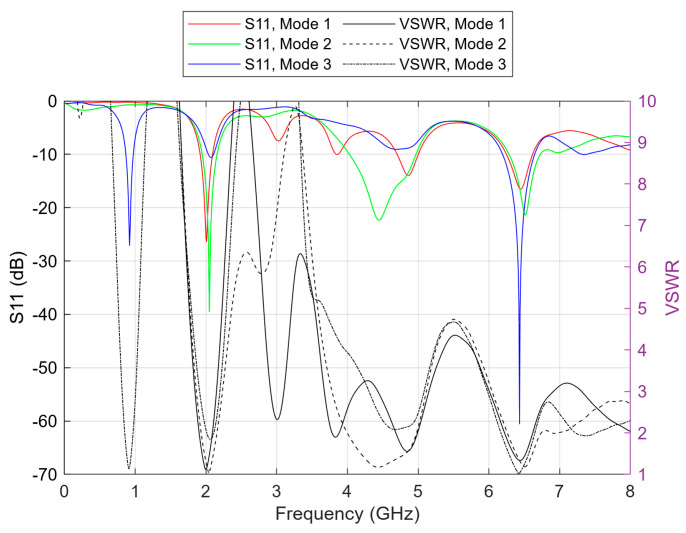
Simulation results of S11 parameter and VSWR for the antenna’s three modes of operation.

In the simulation, the resistance (R) and the capacitance (C) values were selected from the diode’s datasheet graphs, considering the voltage on the diode to be between 0 and 900 mV. L is always selected as 0.7 nH, as defined for the SC-79 diode package. Simulations encompassed the entire 0 to 900 mV range, corresponding to values of R ranging from 1 to 1000 Ω under forward-bias conditions. Notably, three distinct cases stood out, distinguished by the conspicuously low S11 parameter values observed at their primary resonant frequencies. These cases were denoted as modes 1, 2, and 3. Mode 1 corresponds to 0 voltage on the diode, with values C = 0.3 pF and R = 5 kΩ. Modes 2 and 3 correspond to a forward biasing of the diode with no C and R = 160 Ω or R = 4 Ω, respectively. The simulated S11 parameter and VSWR results for these three modes are illustrated in Figure 3.

**Figure 4:**
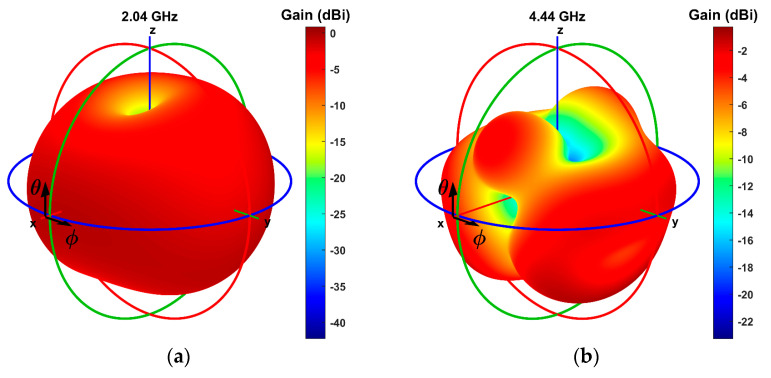

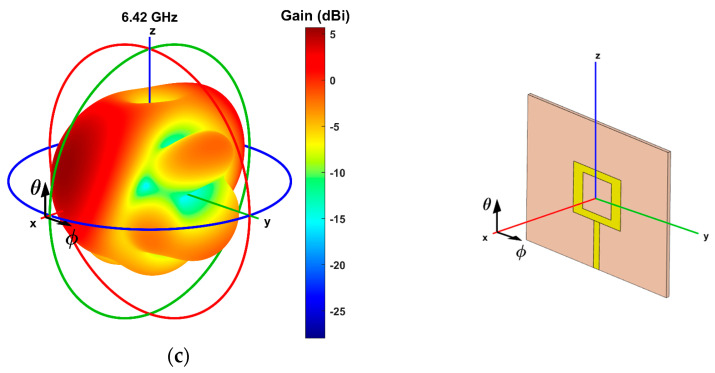
Simulated 3D radiation patterns for frequencies (**a**) 2.04 GHz, (**b**) 4.44 GHz, and (**c**) 6.42 GHz.

As indicated by Figure 3, the primary resonant frequency for mode 1 is 2 GHz, with a return loss (S11) of −26.4 dB, which is about 9.5 dB lower than the next (secondary) resonance seen at 6.4 GHz. VSWR of the 2 GHz resonance for this mode was equal to 1.11. Subsequently, mode 2 shows a better main resonance at 2.04 GHz frequency than mode 1 in the 2 GHz band. It is characterized by a good return loss of −39.5 dB and a VSWR equal to 1.02. Amongst the three modes, this one has the best resonance in the 4 GHz band, at 4.44 GHz specifically. The return loss for this resonance is −22.3 dB, and the VSWR is 1.17. Finally, mode 3’s primary resonance is found at 6.42 GHz, with an excellent return loss of −60.6 dB, in addition to a very low VSWR found at 1.01 for this resonant frequency. The following, secondary, resonance is seen at 0.91 GHz, with a return loss of about 33.5 dB higher than that of the main resonance. Overall, according to the simulation results, the antenna can operate in the 2, 4, and 6 GHz bands. Mode 2 is the most convenient for operation in the 2 and 4 GHz bands at frequencies of 2.04 and 4.44 GHz, while mode 3 is the best for operation in the 6 GHz band at 6.42 GHz. The −10 dB defined bandwidth for the frequencies 2.04, 4.44, and 6.42 GHz is 211, 941, and 452 MHz, respectively. The radiation patterns for these three cases are depicted in Figure 4, where the positive *x*-axis extends towards the front direction of the antenna. The azimuth angle will be referred to as φ, while the elevation angle as θ. Both are 0° at the antenna’s front while 180° at the back.

According to the 2.04 GHz radiation pattern, the maximum gain of the antenna is, in this case, 0.82 dBi, observed at angles θ = 355° and φ = 180°. Furthermore, in front of the antenna, for θ = φ = 0°, the gain is −0.78 dBi. Similarly, the 4.44 GHz pattern shows a maximum gain of −0.31 dBi, at angles θ = 310° and φ = 135°. In front of the antenna, at θ = φ = 0°, the gain is −15.74 dBi. However, the gain can be increased to −3.78 dBi with the antenna’s rotation to an angle of θ = 34° on the elevation plane. Finally, the 6.42 GHz radiation pattern shows a maximum gain of 5.68 dBi, at angles of θ = 20° and φ = 0° while at angles of θ = φ = 0°, it equals 4.13 dBi.

As derived from the simulation, the antenna performs best when operating on mode 3, with a main resonance at 6.42 GHz. It demonstrates the maximum gain, and lowest return loss and VSWR.

**Figure 5:**
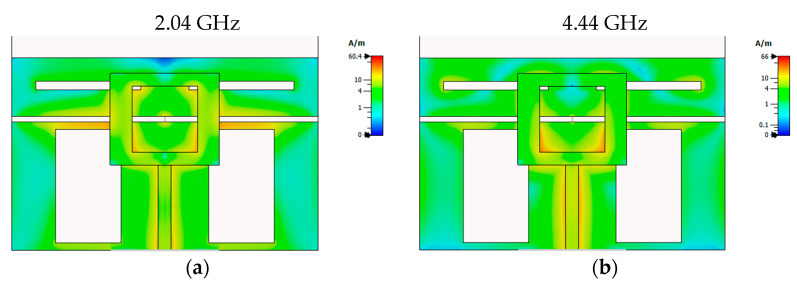

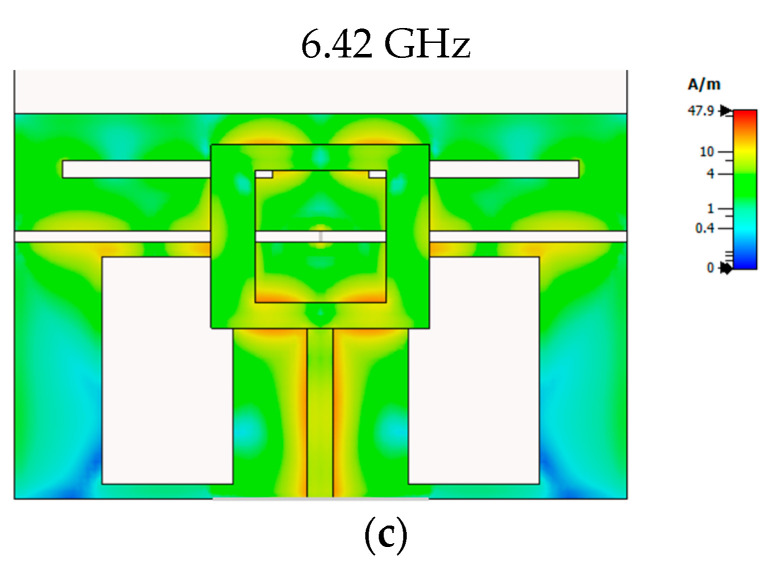
Simulated surface current distribution for frequencies (**a**) 2.04 GHz, (**b**) 4.44 GHz, and (**c**) 6.42 GHz.

Finally, Figure 5 provides a visual representation of the surface current distribution across the antenna for its three primary resonant frequencies: 2.04, 4.44, and 6.42 GHz. It is evident from the figure that, in addition to the patch, the ground plane significantly influences the antenna’s radiation characteristics. Although the current is distributed across the entire antenna surface, there is a pronounced concentration behind the patch. Moreover, in all three cases, substantial contributions to the antenna’s radiation emanate from the edge of the two segments of the ground plane, which are connected via the PIN diode. There are noticeable differences in the surface current distribution for each frequency. These distinctions in the surface current distribution directly translate into discernible differences in radiation patterns among the three frequencies, as observed in Figure 4.

Last but not least, the simulations demonstrate a good radiation efficiency for the antenna across its three primary resonant frequencies. At 2.04 GHz, the radiation efficiency stands at an exceptional 74%, while at 4.44 GHz and 6.42 GHz, it remains notably high at 59% and 54%, respectively. These findings are consistent with typical performance expectations, signifying the absence of significant losses on the antenna.

The simulation results for the three resonant frequencies, where the antenna seems to function optimally, are summarized in Table 2. This summary includes the resonant frequencies, their return loss (S11), the VSWR, the bandwidth as defined on a −10 dB level of return loss, the antenna’s maximum gain for each case, as well as the azimuth and elevation angles where the maximum gain was observed.

## 4. Experimental Results and Discussion

**Figure 6:**
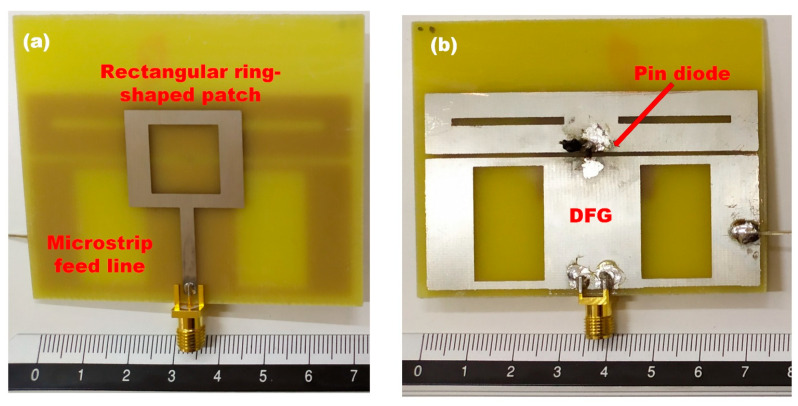
Fabricated multi-frequency reconfigurable patch antenna (**a**) top side and (**b**) bottom side.

The front and the back side of the fabricated multi-frequency reconfigurable patch antenna is shown in Figure 6. For the purpose of providing the biasing voltage to the PIN diode, two pins were soldered on the antenna, one next to the diode’s anode, and the second to the side of the lower part of the ground plane. By varying the voltage on the diode, three modes arise with main resonances in the 2, 4, and 6 GHz bands, as dictated by the simulation results. Mode 1 corresponds to the best performance in the 2 GHz band, mode 2 in the 4 GHz band, and mode 3 in the 6 GHz band. Each mode is characterized by a forward-biasing voltage of 0, 550, and 740 mV, respectively. 

**Figure 7:**
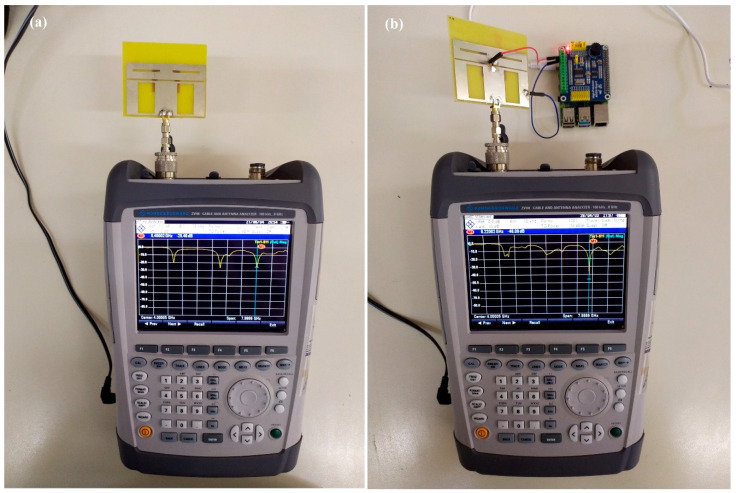
S11 measurement setup for the antenna (**a**) before adding the PIN diode and (**b**) after adding the PIN diode, with the antenna operating in mode 3 (6.22 GHz).

A Rohde & Schwarz ZVH8 Handheld VNA was used to experimentally validate the performance of the multifrequency reconfigurable patch antenna through S11 and VSWR. Figure 7a shows a snapshot of the S11 measurement for the multifrequency antenna before adding the PIN diode, while Figure 7b shows the S11 measurement for the reconfigurable antenna operating under mode 3 (6.22 GHz). The reconfiguration of the antenna is performed remotely through an Android application that changes the excitation voltage on the diode using a Raspberry platform.

It is also essential to mention that, in our setup, we utilized a high-quality DAC as the voltage source for biasing the diode. The DAC’s output has a low impedance of 1 Ohm, ensuring a stable DC bias voltage without significant AC components. Additionally, it provides exceptional isolation from AC signals, about 100 dB. Consequently, while the inclusion of chokes is essential in many cases, it was deemed non-critical for our application.

**Figure 8:**
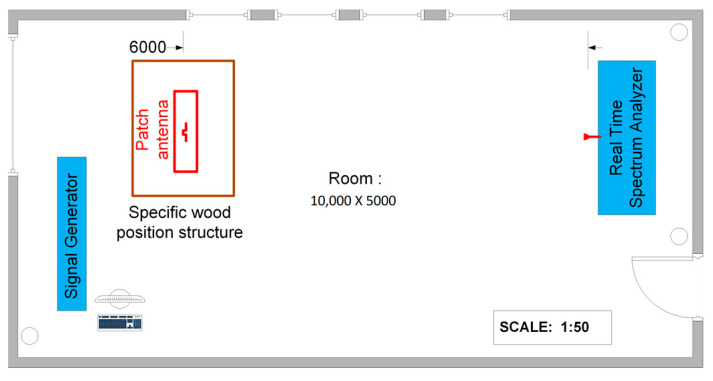
Free-space lab measurement plan.

**Figure 9:**
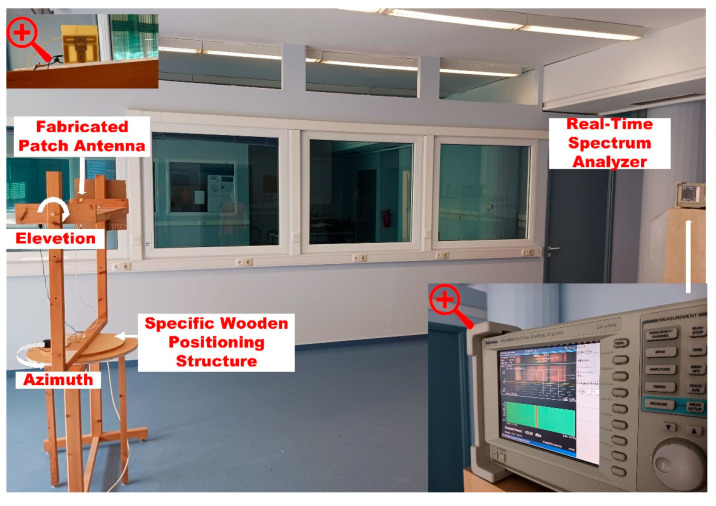
Measurement setup.

The measurements of the antenna radiation pattern took place in a free-space lab environment in the university with dimensions of 5 m width by 10 m length. The free-space lab measurement plan appears in Figure 8. The fabricated prototype patch antenna was placed on top of a specific wooden positioning structure at a height of 1.5 m from the ground and at 6 m was the receiving antenna on a Tektronix RS3408A real-time spectrum analyzer. The fabricated patch antenna was stimulated using a signal generator, transmitting an unmodulated carrier at the frequency of three operation modes. The measurements for each operation mode were conducted at a 10-degree-step resolution, and to optimize the results, they were repeated five times. Each value on the antenna radiation pattern is the average value. Figure 9 shows a photograph of the measurement setup.

**Figure 10:**
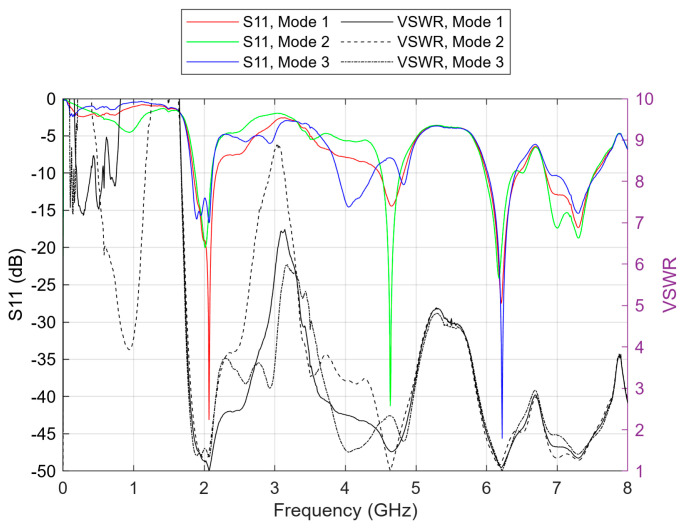
Experimental results of the S11 parameter and VSWR for the three modes of operation.

The S11 and VSWR results are depicted in Figure 10. According to the experimental results in Figure 7 and Figure 8, the antenna shows three excellent resonances in the 2, 4, and 6 GHz bands with a return loss well below −40 dB and a VSWR no higher than 1.02 in all cases. Specifically, the primary resonance for mode 1 is found at 2.07 GHz, with a −43.1 dB return loss, which is 15.6 dB lower than the return loss of a secondary resonance seen at 6.21 GHz. The VSWR at the 2.07 GHz resonance is 1.01, and the −10 dB bandwidth is 327 MHz. Concerning mode 2, it shows a main resonance at 4.63 GHz. It has a return loss of −41.3 dB, 17.1 dB lower than that of a secondary resonance at 6.17 GHz. The value of VSWR, in this case, is 1.02, and the −10 dB bandwidth is 260 MHz. Finally, for mode 3, the primary resonance is found at 6.22 GHz, and the return loss is −45.6 dB, 28.9 dB lower than that of a secondary resonance found at 2.07 GHz. The VSWR of the 6.22 GHz resonance is 1.01, and the bandwidth defined at −10 dB return loss is found at 387 MHz. 

Regarding the truncation of the VSWR plots (Figure 3 and Figure 10), it is important to note that the optimal VSWR value is 1. In practice, real antennas commonly exhibit VSWR values within the range of 1 to 2 at their resonant frequencies. Values exceeding 10 are not displayed in the figures, as they signify a significant portion of the radiation being reflected and not reaching the antenna. This adjustment was made to provide a clearer focus on the region close to 1, which holds greater significance.

**Figure 11:**
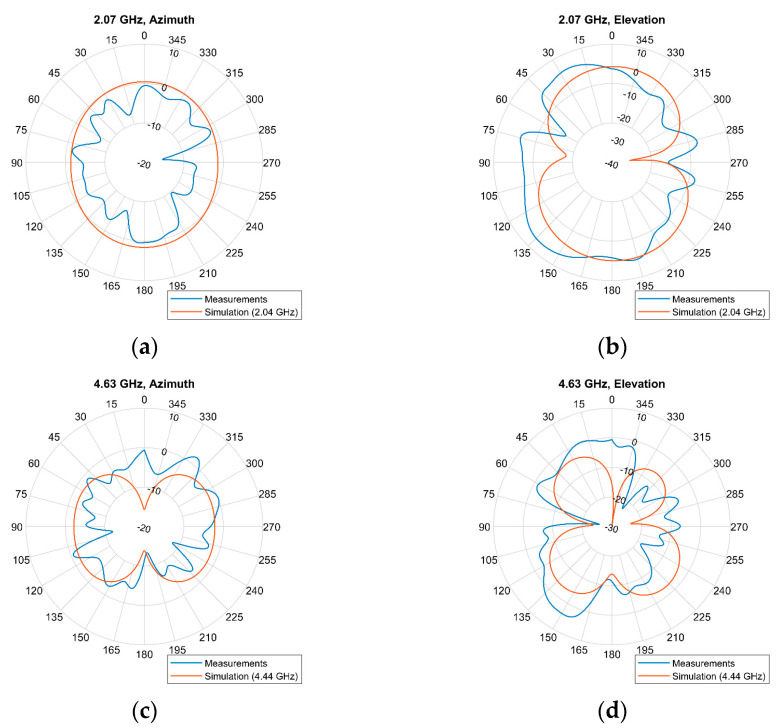

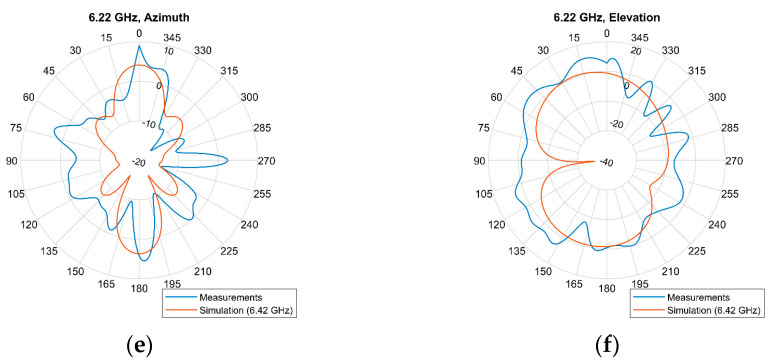
Measured radiation patterns (in blue) on azimuth (**left**) and elevation (**right**) planes for frequencies: (**a**,**b**) 2.07 GHz, (**c**,**d**) 4.63 GHz, and (**e**,**f**) 6.22 GHz, along with the respective simulated radiation patterns (in red) for frequencies 2.04, 4.44, and 6.42 GHz.

Next, the radiation patterns for these three modes at 2.07, 4.63, and 6.22 GHz, on both the azimuth and the elevation planes, are illustrated in Figure 11. Like before, the azimuth angle will be referred to as φ, while the elevation angle is defined as θ. Furthermore, the simulation results at 2.04, 4.44, and 6.42 GHz are also included in the figure.

While the cross-polarization pattern can offer valuable insights into how the antenna interacts with signals of different polarizations, it is excluded (Figure 11) for the sake of conciseness. The information already presented, including S11/VSWR, radiation pattern, gain, and, now, efficiency for the primary resonances, can adequately convey a comprehensive understanding of the antenna’s performance. In turn, according to the 2.07 GHz radiation pattern, the gain of the antenna on the azimuth plane, for elevation angle θ = 0°, has a maximum value of 0.45 dBi for an azimuth angle φ = 176°, at the back of the antenna. As for the forward direction of the antenna, for azimuth and elevation angles φ = θ = 0°, the gain is −0.54 dBi. By rotating the antenna on the elevation plane at an angle θ = 25°, the gain can be improved to the value of 3.81 dBi, while φ = 0°. Moreover, the absolute maximum gain is 5.48 dBi, which is observed at an elevation θ = 140° at the back of the antenna (φ = 180°). Similarly, the 4.63 GHz patterns indicate that on the azimuth plane (θ = 0° or 180°), the highest gain value is 1.92 dBi found at φ = 322°. On the forward direction of the antenna, for φ = θ = 0°, the gain is 0.58 dBi. Furthermore, taking both planes into account, a maximum gain could be equal to 3.97 dBi at an elevation of θ = 152° and azimuth of φ = 180°. Finally, the 6.22 GHz radiation patterns demonstrate a primary lobe with a maximum gain of 9.12 dBi, on the azimuth plane at φ = 0° and θ = 0°. In the condition of φ = 0°, and if the antenna is rotated to an angle θ = 12° on the elevation plane, then the gain can be further increased up to 12.79 dBi.

The experimental results indicate that the antenna operates almost equally well in all three bands of 2, 4, and 6 GHz, at the resonant frequencies of 2.07, 4.63, and 6.22 GHz. However, at 6.22 GHz, a much higher gain can be attained. A summary of the results, derived from the network analyzer measurements and the radiation patterns, is shown in Table 3. That includes the resonant frequency, return loss (S11), VSWR, the bandwidth as defined on a −10 dB return loss level, the maximum attainable gain for the three main resonances, as well as the azimuth and elevation angles where observed. 

As compared to the simulation results, some slight deviations are observed. In the simulation, the three main resonances were located at 2.04, 4.44, and 6.42 GHz, while, on the fabricated antenna, they were found at 2.07, 4.63, and 6.22 GHz. The deviation between the three cases is 30, 190, and 200 MHz, respectively, or 1.4%, 4.2%, and 3.1%.

Also, the experimental results show a better functionality of the antenna at 4.63 GHz, than the one suggested by the simulation at 4.44 GHz. Furthermore, the radiation patterns of the fabricated antenna show better gains than those seen in the simulation. For the 2, 4, and 6 GHz bands, the maximum simulation gain was 0.58, 1.75, and 5.64 dBi, respectively, while the corresponding measured gains were found as 5.48, 3.97, and 12.79 dBi, which are 4.9, 5.72, and 7.15 dB higher than those suggested by the simulation. The deviations between the simulation and the experimental results are overall insignificant, except for the simulated and measured gain. These deviations were expected since many factors were not accounted for in the simulation. Such factors are the effects of solder used for the SMA connector, the PIN diode, and the effects of the pins used for applying voltage on the diode. Also, the antenna was tinned to avoid oxidation, which could also have some effect on its functionality. But more importantly, the deviations could be due to the R and C values selected for the diode’s equivalent circuit in the simulation. Both these parameters, but mostly R, are frequency-dependent. Because the simulation tool does not support nonlinear elements in the simulations, they were both considered constant over the whole simulated frequency range, from 0 to 8 GHz. Furthermore, the graphs in the diode’s datasheet describe these values for frequencies only up to 2 GHz. Thus, the R and C behavior for higher frequencies was considered to be similar, which might not be the case. This probably is the reason that the deviation appears to be minimal at the 2 GHz band. Finally, the deviations in the radiation patterns, and primarily in the gain, might be due to reflections occurring in the vicinity of the antenna, or due to the prime resonances of the fabricated antenna not being identical to those indicated by the simulation.

The compact patch re-configurable antenna is controlled by a single PIN diode, in contrast to several other works that require multiple diodes. The antenna has three modes of operation with a total bandwidth of 974 MHz over prevailing tuning in the three frequencies. Furthermore, it has a low cost, volume, and weight and can be easily implemented even in a lab-based environment. The antenna also achieved quasi-omnidirectional radiation patterns that make it ideal for wireless applications where omnidirectional coverage is usually required.

### Comparative Study

As we have already mentioned, there are several techniques to design and implement a reconfigurable antenna. The comparative study is based on antennas being similar to the one presented in this work. They are all printed on an FR-4 substrate and utilize PIN diodes to attain the reconfiguration. Table 4 shows the key features of the antenna presented in each work, specifically, the dimensions of the antenna, the number of PIN diodes used, and the place of their installation, i.e., whether it is on the patch and/or on the ground plane. The table also includes the operating frequencies of each antenna, as well as its maximum gain, and whether it assumes the advantages of Defected Ground Structure (DGS) or not.

Table 4 clearly illustrates the prevalence of employing PIN diodes in prior studies. Multiple diodes were used in the majority of them. In contrast, this study distinguishes itself by utilizing a single PIN diode. Conventionally, reconfigurable antennas tap into the two states of the PIN diodes, ON (forward bias) and OFF (reverse bias). Notably, the antenna proposed herein demonstrates three distinctive modes of operation (based on the two states of the diode). It is worth mentioning that varying the forward biasing voltage can result in multiple modes. The use of a singular PIN diode and the low forward biasing voltage it requires offer convenience, and the process of reverting between the three modes can be very straightforward to automate with devices such as a microcontroller. Moreover, this work employed a Defected Ground Structure (DGS), a feature scarcely used in previous works. By and large, the proposed antenna exhibits an omnidirectional radiation pattern across all frequencies, along with low return loss, a broad bandwidth, and exceptionally high maximum gain. Lastly, the compact size of the antenna makes it convenient for applications where portability is important and where reduced space is a key matter such as in military applications.

## 5. Conclusions

In this paper, a novel reconfigurable antenna operating in the 2, 4, and 6 GHz bands is presented. Reconfiguration is achieved using a single PIN diode. The antenna was simulated and implemented. The fabricated antenna exhibits three modes of operation with resonances at 2.07, 4.63, and 6.22 GHz. The three modes were defined by different values of the forward biasing voltage applied to the diode, in the range of just zero to 900 mV. The return loss is well below 40 dB for the primary resonance in every mode, and the VSWR is minimal, 1.01–1.02. The experimental results of the radiation patterns suggest that the gain is maximum at the bottom side of the antenna and equal to 5.48 dBi when operating in the 2 GHz band, 3.97 dBi on the bottom side when operating in the 4 GHz band, and 12.79 dBi in front of the antenna when operating in the 6 GHz band. If the antenna works for transmission or reception solely in the forward direction, it should be rotated toward the front by an elevation angle between 12° and 25° for maximum gain. In this case, depending on the rotation angle, the maximum gain which can be attained in the 2, 4, and 6 GHz band resonances is 3.81, 0.36, and 12.79 dBi, respectively. Finally, the fabricated antenna demonstrated better functionality in the 4 GHz band than suggested by the simulation, while the measured gain was much higher in all cases. Overall, the deviation between the simulation and the experimental results was negligible.

## Figures and Tables

**Table 1:** The antenna’s design parameters.

Design Parameter	Dimensions (mm)	Design Parameter	Dimensions (mm)
Ws	70	Lsl1	26
Ls	60	Wsl2	24
Wp1	25	Lsl2	2
Lp1	21	Lg1	29.35
Wp2	15	Lg2	13.35
Lp2	15	a	1.675
Wf	3	b	1.3
Lf	19.5	c	5.68
Wsl1	15	d	5.5

**Table 2:** Simulation results summary for the three primary resonant frequencies in the 2, 4, and 6 GHz bands.

Frequency (GHz)	S11 (dB)	VSWR	−10 dB Bandwidth (MHz)	Max Gain (dBi)	Azimuth and Elevation of Max Gain
2.04	−39.5	1.02	211	0.82	φ = 180°, θ = 355°
4.44	−22.3	1.17	941	−0.31	φ = 135°, θ = 310°
6.42	−60.6	1.01	452	5.68	φ = 0°, θ = 22°

**Table 3:** Experimental results summary for the three primary resonant frequencies in the 2, 4, and 6 GHz bands.

Frequency (GHz)	S11 (dB)	VSWR	−10 dB Bandwidth (MHz)	Max Gain (dBi)	Azimuth and Elevation of Max Gain
2.07	−43.1	1.01	327	5.48	φ = 180°, θ = 140°
4.63	−41.3	1.02	260	3.97	φ = 180°, θ = 152°
6.22	−45.6	1.01	387	12.79	φ = 0°, θ = 12°

**Table 4:** Comparison between the proposed antenna and those of other, related works.

Ref.	Dimensions (mm^2^)	No. of Diodes	Diode Location	Operating Frequencies (GHz)	Max Gain (dBi)	DGS
[31]	25 × 25	2	Patch and Ground Plane	4.80, 5.32, 6.01, 6.22, 6.41	3.85	Yes
[32]	16 × 22	2	Resonator	2.09, 2.15, 3.16, 4.3, 4.8, 5.2, 5.3	2.5	No
[14]	66 × 58	1	Patch	2.4, 3.8, 5.3	5.34	No
[33]	44 × 14	2	Radiating Element	0.84, 2.12	2.12	No
[34]	23 × 31	3	Patch and Ground Plane	3.1, 6.8, 2.5–4.2, 6.2–7.4	4.6	No
[24]	32 × 40	4	Patch	1.8, 2.1, 2.6, 3.5, 5, 5.6, 6.4, 6.5	3.6	No
[35]	29 × 34	5	Resonator	Multiple between 1.74 and 4.84 GHz	3.86	No
[36]	30 × 30	4	Patch	2.52, 2.68, 3.49, 3.58	4.46	No
[37]	46 × 32	12	Patch	1.95–2.30, 2.36–4.17, 4.52–5.39	4.67	No
[38]	30 × 20	3	Patch	2.1, 2.6, 3.5, 5.0, 6.2, 6.4, 3.51–8.51	2.5	No
[39]	58 × 50	2	Ground Plane	1.750, 1.7750, 1.815, 1.610	1	Yes
[40]	80 × 50	2	Patch	0.915, 0.433, 0.868	2	No
[41]	50 × 46	5	Ground	1.777, 1.909, 1.986, 2.151, 2.302, 2.421	3.23	No
This Work	70 × 60	1	Ground Plane	2.07, 4.63, 6.22	12.79	Yes

## Data Availability

The data that support the findings of this study are available from the corresponding authors, upon reasonable request.
